# Early diagnosis of HTLV-1-associated myelopathy (HAM/TSP) in HTLV-1 carrier clinic

**DOI:** 10.1186/1742-4690-11-S1-P29

**Published:** 2014-01-07

**Authors:** Toshio Matsuzaki, Ryuji Kubota, Hiroshi Takashima, Shuji Izumo

**Affiliations:** 1 Divisions of Molecular Pathology, Center for Chronic Viral Diseases, Kagoshima University, Japan; 2 Department of Neurology and Geriatrics, Kagoshima University, Japan

## 

Early diagnosis and medical intervention are important for functional prognosis of HAM/TSP patients. In order to find out patients with early stage of HAM/TSP, we started HTLV-1 carrier clinic in the endemic area, Kagoshima, Japan, in 1999. Till the end of 2012, 407 persons have visited the clinic as first time-visitors of HTLV-1 carriers, and 6 cases were diagnosed as early stage of HAM/TSP. These 6 cases are 1 male and 5 females, 20-55 years old (mean=41.8) at the diagnosis, no history of blood transfusion, and 4 cases have family history of HAM/TSP. They could run and had no subjective symptoms on motor function, but all had subjective symptoms either dysesthesia/pain or urinary disturbances. Physical examination demonstrated hyper-reflexes of lower extremities with mild spasticity, positive Babinski signs, and decreased sweating of lower trunk and legs. Laboratory test showed positive anti-HTLV-1 antibodies in both sera and CSF, and proviral loads in the blood were high (386-2181 copies/104 PBMC) in all cases. Two cases were treated with steroids and their urinary disturbances were improved. After 2 years of follow up, their symptoms remained unchanged and decrease of proviral load was obtained in one case. These experiences in HTLV-1 carrier clinic indicate that early diagnosis of HAM/TSP is possible by careful medical checking of HTLV-1-positive individuals. Decrease of sweating in lower body, and hyper-reflexes of legs with typical Babinski sign are important signs for early diagnosis. Treatment intervention at early stage of the disease might improve functional prognosis of HAM/TSP patients.

